# Three-Dimensional Image of A Communicating Uterus

**Published:** 2013-09-18

**Authors:** Firoozeh Ahmadi, Hadieh Haghighi

**Affiliations:** Department of Reproductive Imaging at Reproductive Biomedicine Research Center, Royan Institute for Reproductive Biomedicine, ACECR, Tehran, Iran

Recent advances in reproductive medicine have created a demand for more accurate and safe imaging modalities before assisted reproductive treatment (ART) (1). Gradually, the important role of three-dimensional (3D) ultrasonography in the diagnosis of uterine congenital anomalies has been proved (2-4).

**Fig 1:**
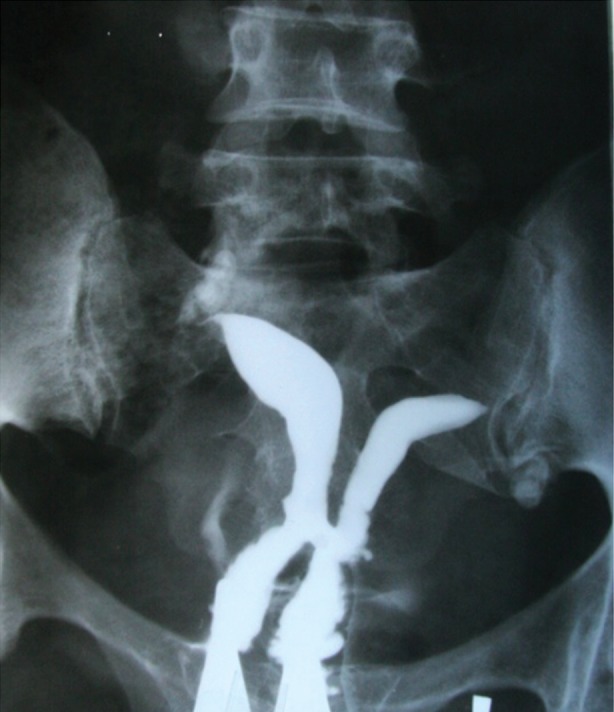
Hysterosalpingogram demonstrates a communicating septate uterus, cervix duplex.

**Fig 2:**
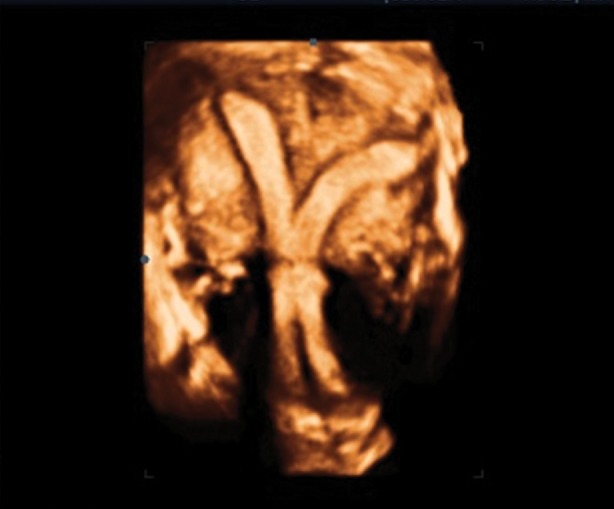
3D image of a communicating septate uterus, which is quite similar with hysterosalpingography image.

The aim of this article is to evaluate a communicated uterus through 3D image, while to compare this result with quite similar images of this patient with hysterosalpingography (Fig1).The patient’s past medical history indicated three first -trimester miscarriages. Therefore, she underwent a hysterosalpingogram (HSG), and was also referred to 3D ultrasound as part of her infertility treatment (before ART). Figure 2 shows an image taken through a 3DXI (ACCUVIX XQ, Medison, South Korea) ultrasound with a 6.5-MHz transvaginal probe equipped with three-dimensional imaging. The uterus was examined systematically .On the coronal view, a long septum divides the cavity and cervix into two parts, and between the divided parts, a connection in isthmus can easily be identified. Congenital malformation of uterus is caused by a numerous anomalies during embryogenesis. The American Society for Reproductive Medicine (ASRM) has classified müllerian duct anomalies (MDAs) to provide substantial assistance in the clinical application of infertility and preoperative decision (5). A communicating uterus, as a rare type of müllerian duct anomaly, does not fall into the classification system of ASRM (6). An alternative embryological deficiency, reviewed by Musset’s classification, describes this anomaly (6). According to this theory, fusion first occurs at the level of the uterine isthmus, and simultaneously, proceeds into the both directions of caudal and cephalad. Later, uterine corpus and cervix are formed by midline resorption initiating at the isthmus, followed by rapid cellular bidirectional resorption of septum. It is not clear whether this is the mechanism for normal müllerian development, or a developmental failure which is unique to other rare anatomical divergence (7). Recent developments in three-dimensional ultrasonography can greatly strengthen diagnostic potential of female reproductive tract anomalies, and also, should be considered as first-line examination. It has the advantages of an easy, inexpensive, reproducible and noninvasive tool for analyzing of morphologic anatomy, which deserves more attention by gynecologists (2). In certain cases, MRI, hysteroscopy and laparoscopy are more effective techniques than others. (3).
